# Potent Natural Antioxidant Carveol Attenuates MCAO-Stress Induced Oxidative, Neurodegeneration by Regulating the Nrf-2 Pathway

**DOI:** 10.3389/fnins.2020.00659

**Published:** 2020-06-26

**Authors:** Imran Malik, Fawad Ali Shah, Tahir Ali, Zhen Tan, Abdullah Alattar, Najeeb Ullah, Arif-ullah Khan, Reem Alshaman, Shupeng Li

**Affiliations:** ^1^Riphah Institute of Pharmaceutical Sciences, Riphah International University, Islamabad, Pakistan; ^2^State Key Laboratory of Oncogenomics, School of Chemical Biology and Biotechnology, Shenzhen Graduate School, Peking University, Shenzhen, China; ^3^Department of Comparative Biology and Experimental Medicine, Faculty of Veterinary Medicine, Hotchkiss Brain Institute, Cumming School of Medicine, University of Calgary, Calgary, AB, Canada; ^4^Health Management Center, Shenzhen University General Hospital, Shenzhen University Clinical Medical Academy, Shenzhen, China; ^5^Department of Pharmacology and Toxicology, Faculty of Pharmacy, University of Tabuk, Tabuk, Saudi Arabia; ^6^Institute of Basic Medical Sciences, Khyber Medical University, Peshawar, Pakistan

**Keywords:** middle cerebral artery occlusion, carveol, all-trans retinoic acid, antioxidant enzymes, Nrf2 pathway

## Abstract

Ischemic stroke is a severe neurological disorder with a high prevalence rate in developed countries. It is characterized by permanent or transient cerebral ischemia and it activates syndrome of pathological events such as membrane depolarization, glutamate excitotoxicity, and intracellular calcium buildup. Carveol is widely employed as anti-inflammatory and antioxidant in traditional Chinese medicine. In the present study, the neuroprotective effects of post-treated carveol were demonstrated against transient middle cerebral artery occlusion (MCAO) induced focal ischemic cerebral injury. Male Sprague Dawley (SD) rats were subjected to two different experimental protocols to determine the dose and effects of carveol, and to demonstrate the underlying role of the nuclear factor E2-related factor (Nrf2) pathway. Our results showed that MCAO induced marked neuronal injury in the ipsilateral cortex and striatum associated with higher inflammatory cytokines expression, along with apoptotic markers such as caspase-3 and the phosphorylated *c*-Jun *N*-terminal kinase (JNK). Furthermore, MCAO induced a marked increase in oxidative stress as evidenced by high lipid peroxidase (LPO) content accompanied by the depressed antioxidant system. Carveol significantly reversed the oxidative stress and downregulated inflammatory cascades by enhancing endogenous antioxidant mechanisms including the Nrf2 gene, which critically regulates the expression of several downstream antioxidants. Further, to determine the possible involvement of Nrf2 in carveol mediated neuroprotection, we antagonized Nrf2 by all-*trans* retinoic acid (ATRA), and such treatment abrogated the protective effects of carveol accompanied with exaggerated neuronal toxicity as demonstrated by higher infarction area. The target effects of carveol were further supported by molecular docking analysis of drug–protein interactions. Together, our findings suggest that carveol could activate endogenous master anti-oxidant Nrf2, which further regulates the expression of downstream antioxidants, eventually ameliorating MCAO-induced neuroinflammation and neurodegeneration.

## Introduction

Ischemic stroke is a neurological disorder with a high prevalence in developed countries. Ischemic stroke has a higher incidence ratio of about (80%) as compared to other types and occurs due to occlusion in the middle cerebral artery (MCA) (Dirnagl et al., 1999). In ischemic stroke, either permanent or briefly transient hindrance of blood flow occurs which leads to decreased oxygen and nutrient supply to the brain. Tissue plasminogen activator (tPA) is the only FDA approved drug for stroke which acts via vascular recanalization. Ischemic injury can cause a varying degree of cellular damage marked by complex events of signaling cascades (Dirnagl et al., 1999). Immediately after the stroke, ATP and energy production are reduced along with ionic imbalance, which sequentially leads to membrane depolarization and calcium overload. Furthermore, excessive release of glutamate enhances the influx of calcium ions and triggers downstream inflammatory and apoptotic proteins (Barone and Feuerstein, 1999; Wang Q. et al., 2007) along with rapid activation of glia cells (Chung and Benveniste, 1990). This, mingled with the vascular and tissue damage incurred by early mitochondrial dysfunction and oxidative phosphorylation, trigger the release of reactive oxygen species (ROS) and pro-inflammatory mediators such as interleukin-1 (IL-1β), tumor necrosis factor-alpha (TNF-α), and interleukin-6 (IL-6), eventually leading to cellular damage as well as peroxidation of membranous protein and cytoplasmic organelles (Vila et al., 2000). Neuro-inflammation remains the more prominent pathological hallmark of ischemic stroke as it starts hours after ischemia and disseminates to other biochemical cascades for secondary damages. Combating neuro-inflammation is, therefore, an attractive strategy to prevent MCA occlusion (MCAO) induced degeneration. Several lines of evidence and comprehensive literature review enlighted that activation of endogenous and master anti-oxidant Nrf2 (nuclear factor E2-related factor or nuclear factor erythroid 2 or p45-related factor 2) plays an important role in the impaired cellular homeostasis via responding to endogenous and exogenous stressors/insults in different diseases (Suzuki and Yamamoto, 2015; Javeed et al., 2017; Vomund et al., 2017; Liu et al., 2019). Consistent reports reiterated the protective role of Nrf2 in the brain and metabolic disorders (Ramsey et al., 2007; Dinkova-Kostova et al., 2018; Sivandzade et al., 2019). Nrf2 is ubiquitously present in the cytoplasm as a dimer with Keap1 (Nrf2-Keap1), whereas Keap1 inhibits or suppresses Nrf2 activation. Upon activation by stress or exposure to ROS, Nrf2 translocates to the nucleus to activate antioxidant machinery and negatively regulates downstream pro-inflammatory mediators such as NF_*K*_B, and inflammatory cytokines (TNF-α and COX2) (Lee and Johnson, 2004; Hennig et al., 2018). Most importantly, Nrf2 activation and its associated signalings are neuroprotective in ischemic and other brain injuries in various pre-clinical animal models (Hong et al., 2010; Jiang et al., 2017; Zhang et al., 2018; Duan et al., 2019; Shah et al., 2019a). Thus, Nrf2 dependent signaling pathway presents an appropriate therapeutic target to cope against a variety of insults including cerebral ischemia.

Natural products have been exploited as a repository for novel therapeutic identification for decades and thus attracted considerable attention as a source of potential therapeutic agents. This can be attributed to its antioxidant nature consistently validated in experimental models by many researchers. Our group is also aimed to explore the natural-based compounds to prevent and treat ischemic associated detrimental effects (Sung et al., 2012; Gim et al., 2013; Shah et al., 2014, 2019a). Herein, we hypothesized that natural derived substance, carveol act as potent anti-oxidant and prevent MCAO induced brain degeneration. Carveol, a natural monocyclic monoterpenoid abundantly present in the essential oil of orange peel, dill, and caraway seeds (De Carvalho and Da Fonseca, 2006). It is found in caraway, mandarin (*Citrus reticulata*), blackcurrant berries, black tea, and dill (Crowell et al., 1992). The caraway based medicinal plants have been useful in the management of various diseases (Mahboubi, 2019). Carveol has been reported in traditional Chinese medicine as an antispasmodic, carminative, astringent, and further use/used for indigestion and dyspepsia (Sachan et al., 2016). It also demonstrated antioxidant, anti-hyperlipidemic, and anti-inflammatory activities in the liver (Johri, 2011). Furthermore, carveol is equally potent to inhibit COX2 activation and its anti-inflammatory effects are comparable to that of aspirin (Kawata et al., 2008). Nevertheless, as per our knowledge and literature survey, there is no study on carveol in neurodegenerative diseases and ischemic associated brain degeneration. The present study aims to evaluate whether carveol effects on neuroinflammation and oxidative stress could eventually account for cellular protection. If so, the potential molecular and cellular mechanisms underlying these effects need to be further delineated. Results obtained will not only help us to understand the relationship of cascading mechanisms that eventually lead to cell death, but also provide a clue as to the potentials of Nrf2 targeting therapeutics.

## Materials and Methods

### Chemicals and Reagents

Mouse monoclonal anti-HO1 (SC-136960), rabbit polyclonal anti Nrf2 (SC-722), mouse monoclonal anti-p-JNK (SC-6254), mouse monoclonal anti-COX-2 (SC-514489), mouse monoclonal anti-p-NFκB (SC-271908), mouse monoclonal anti-Bcl2 (SC-7382), mouse monoclonal anti-caspase-3 (SC-56053), ABC Elite kit (SC-2018), Ultra Cruz mounting media (SC-516212), mouse anti-rabbit IgG-R (SC-2492), and 3,3’-diaminobenzidine peroxidase (DAB) (SC-216567) were purchased from Santa Cruz Biotechnology. The horseradish peroxidase-conjugated secondary antibodies were obtained from Abcam (ab-6789, ab-6721). p-NF-κB Elisa kit (Cat # SU-B28069) and Nrf2 Elisa kit (cat. no. SU-B30429) were purchased from (Shanghai Yuchun Biotechnology, China). HO-1 Elisa kit (cat. No. E-EL-R0488), and TNF-α Elisa kit (cat. No. E-EL-R0019) were got from Elabscience. PBS tablets and proteinase K were obtained from (MP Bio, United States). Formaldehyde, hydrogen peroxide (H_2_O_2_), reduced glutathione (GSH), trichloroacetic acid (TCA), 1-chlor-2,4-dinitrobenzene (CDNP), *N*-(1-naphthyl)ethylenediamine dihydrochloride, 5,5′-dithio-bis-(2-nitrobenzoic acid) (DTNB), and carveol (catalog No: 192384 a mixture of isomers, with 97% purity) was purchased from (Sigma-Aldrich, United States). All-*trans* retinoic acid (ATRA) of the highest analytical grade (99% HPLC) was purchased from the local pharmaceutical industry (GSK).

### Animal Grouping and Drug Treatment

**FIGURE 1:**
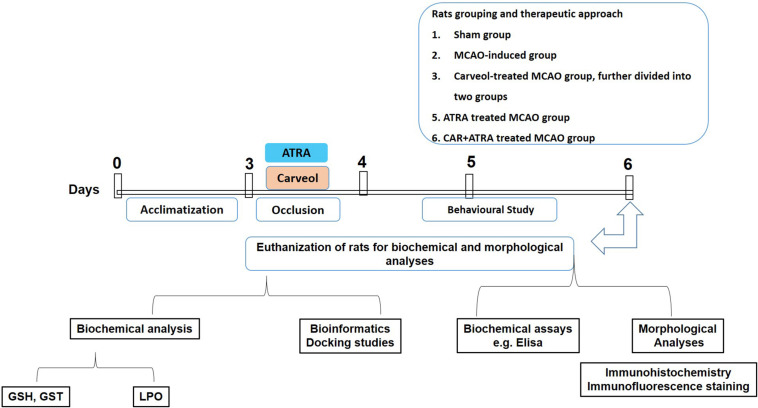
Schematic representation of the in vivo study design. Rats acclimatization, MCAO surgery, drug administration, neurobehavioral studies, euthanization of rats for tissue collection to carry out biochemical analysis, i.e., GST, GSH, catalase and lipid peroxidation (LPO), morphological analysis (2,3,4-triphenyl tetrazolium chloride staining, hematoxylin, and eosin staining and immunohistochemistry), and biochemical assay (enzyme-linked immunosorbent assay, ELISA).

Adult male Sprague–Dawley rats weighing 230–260 g, 7–10 weeks were obtained from Riphah International University, Islamabad. The experimental animals were housed at Laboratory Animal Research Center, Riphah International University, under 12 h light/12 h dark cycle at 18–22°C and had free access to diet and tap water throughout the study. The experimental procedures were set in such a way to minimize rats suffering. All experimental procedures were carried out according to the protocols approved by the Institutional Animal Care and Use Committee of Riphah Institute of Pharmaceutical Sciences (ref no.: REC/RIPS/2018/06) and were strictly adhered to the approved protocols, in addition, to ARRIVE guidelines with few exceptions. We did not apply human endpoints for euthanizing the rats as the ischemic stroke (MCAO model) is the most stressful invasive procedure and in which limited mobility with severe suffering is an established documented protocol, and our group was more interested in rats that survive this period. The exclusion criteria include animals showing no depressed signs or alteration in movements after awakening from anesthesia. By this, we did not euthanize any rats until 72 h of the ischemic period. We applied all laboratory procedures to minimize rat sufferings such as heating pad, sterilization, and fluid replenishment with normal saline. The rats were randomly divided into the following six groups (*n* = 17/group, Figure 1):

1. Vehicle treated control group/Sham; rats in this group received normal saline (containing 5% DMSO).
2. Transient MCAO (t-MCAO) group. MCAO was carried out for 120 min followed by 72 h reperfusion.
3. CR + MCAO group: carveol dissolved in a mixture of normal saline and 5% DMSO and was administrated at 30 min, 24, 48, and 72 h after the MCAO (at doses of 10 and 20 mg/kg).
4. ATRA + MCAO group: single-dose (5 mg/kg) of ATRA dissolved in normal saline (containing 5% DMSO) was administered intraperitoneally 30 min before the induction of ischemia.
5. CR + ATRA + MCAO group: ATRA was administrated as mentioned and carveol (20 mg/kg) was administered intraperitoneally 30 min, 24, 48, and 72 h after ischemia.

All rats were sacrificed 6 h after the last treatment. A total of 10 animals died during the experimental procedures including three from the MCAO group, three from low dose carveol (10 mg/kg), one from high dose carveol (20 mg/kg), two from CR + ATRA + MCAO group and one from the sham group, which we further adjusted by supplementing more animals. The reported reason for this mortality is edema formation, BBB leakage, and hypothalamic shutdown (Neumann-Haefelin et al., 2000). The ethics committee is mostly aware of the mortality in experimental setup particularly in this model, as we constantly engaged them for our work.

### The MCAO Surgery

The animals were anesthetized with an I/P administration of a cocktail of xylazine (9 mg/kg) and ketamine (91 mg/kg). The body core temperature was maintained using a heating pad. MCAO was carried out as previously described (Ali et al., 2020; Park et al., 2020; Shah et al., 2016). Briefly, the right common carotid artery (R-CCA) and its bifurcating branches; internal and external carotid arteries were exposed after a midline cervical incision. Superior thyroid artery and the occipital artery, small protrusions from the external carotid artery were identified and knotted with a thin black (6/0) silk and eventually ligated to allow free movement of the external carotid artery. The external common carotid artery was then tied with silk (6/0) near the hyoid bone above the ligated superior thyroid artery and immediately incised near the bifurcating point. Throughout this procedure, extra care was exercised to avoid excessive bleeding. A blue nylon filament (3/0) with a blunted round end was inserted through the pierced opening of the external carotid artery and advanced into the internal carotid artery up to 18–19 mm (depending upon the weight and age of the rat) till the origin of the MCA, whereas a small resistance to the advancement of nylon indicated occlusion of MCA. The nylon is then tied in place with the lumen of the external carotid artery, and the skin was then sealed. All animals were subjected to carbon dioxide (CO_2_) euthanasia for sample collection. The sham group was exposed to similar measures but with no nylon insertion. The only lacking in this method is the absence of the Doppler effect and relative blood flow measurement, though we constantly do the occlusion with suitable experimental skills. The exclusion criteria include animals showing no depressed signs or alteration in movements after awakening from anesthesia. No significant adverse effects were observed in drug-treated animals as pilot toxicity studies indicated a significantly greater dose.

### Neurobehavioral Test

The rats were handled gently and adequately trained at least 3 days before the experimentation. The behavioral studies were conducted at 24, 48, and 72 h post-surgery. For the determination of sensorimotor function, modified 28-point neuro-scoring was conducted (Shah et al., 2019b). The modified 28-point neuro-scoring includes 11 tests having accumulative of 28 score: (1) circling (maximum four points), (2) motility (maximum three points), (3) general condition (maximum three points), (4) righting reflex when placed on the back (maximum one point), (5) paw placement of each paw onto a tabletop (maximum four points), (6) ability to pull self-up on a horizontal bar (maximum three points), (7) climbing on an inclined platform (maximum three points), (8) grip strength (maximum two points), (9) contralateral reflex (maximum one point), (10) contralateral rotation when held by the base of the tail (maximum two points), and (11) visual forepaw reaching (maximum two points). The score ranges from 0 to 28, 0 indicating severe impairment, and 28 for no neurological damage.

### Brain Water Content

Brain edema was quantitated as described previously. Briefly, animals were decapitated under anesthesia and brain samples were removed. The whole-brain was immediately weighed on an electronic analytical balance to obtain the wet weight. Brain samples were then dried at 120°C for 6–8 h to obtain the dry weight. The formula used was as follows:

(wet⁢weight-dry⁢weight)/wet⁢weight×100.

### Staining and Histological Preparation

After being evaluated for neurological deficits, rats were decapitated under anesthesia, and brain tissues were carefully removed. A total of 2-mm thick coronary sections were cut from the frontal lobe with a sharp blade. Slices were incubated in 2% 2,3,5-triphenyl tetrazolium chloride (TTC) for 10 to 20 min in a water bath at 37°C until a thorough demarcation was observed, and then fixed in 4% paraformaldehyde solution and photographed (Sung et al., 2016; Shah et al., 2018). The infarct area was then measured with the ImageJ program and expressed as a percentage to the total area.

To compensate for brain edema, the percent corrected brain infarction was calculated as follows:

Correctedinfarctarea=[lefthemispherearea-(righthemispherearea-infarctarea)]/100

After photographing, these thick coronal sections were embedded in paraffin, and 4-μm thin coronary sections were made by using a rotary microtome. The slides were de-paraffinized in xylene and rehydrated in graded alcohol and proceeded to the following staining techniques.

### Hematoxylin Eosin (H&E) Staining

Tissue sections on coated slides were de-paraffinized with absolute xylene (100%), rehydrated with ethyl alcohol (from 100 to 70%). The slides were rinsed with distilled water and immersed in hematoxylin for 10 min. After due time, the slides were traced for nuclear staining, and if the staining were not satisfactory, the hematoxylin time was further increased. The slides were then kept under running water in a glass jar for 10 min and treated with 1% HCL and 1% ammonia water as previously reported (Gim et al., 2015). The slides were added to the eosin solution for 5–10 min. Slides were then rinsed in water and air-dried for some time. The dried slides were dehydrated in graded ethyl alcohol (70, 95, and 100%). The slides were cleared with xylene and were mounted with a glass coverslip. The slides were pictured with a light microscope (Olympus, Japan) and analyzed by ImageJ, a computer-based program. The number of images per slide was five per group while focusing specifically on vacuoles formation, the number of surviving neurons, and infiltrations (Shah et al., 2018). The TIFF images were optimized to the same threshold intensity for all groups (Sung et al., 2016).

### Immunohistochemical Analysis

Immunohistochemical staining was performed as previously described with minor modifications (Shah et al., 2019c; Iqbal et al., 2020). The slides were processed for the antigen retrieval step (enzymatic method), then washed with PBS. The endogenous peroxidase was quenched by applying 3% hydrogen peroxide (H_2_O_2_) in methanol for 10 min. The slides were incubated with 5% normal goat serum containing 0.1% Triton X-100. After being blocked, the slides were incubated overnight in mouse anti-phosphorylated-*c*-Jun *N*-terminal kinase (p-JNK), mouse monoclonal anti-(COX-2), mouse monoclonal anti (HO-1), mouse monoclonal anti (Nrf2), mouse anti-p-nuclear factor-κB (NF-κB), mouse monoclonal anti-Bcl2, mouse monoclonal anti-caspase-3 (dilution 1:100, Santa Cruz Biotechnology, United States). The following morning, after being washed with 0.1-M PBS, the slides were incubated in biotinylated secondary antibodies (dilution 1:50) according to the origin of the primary antibody and serum used, then incubated with ABC reagents (SCBT, United States) for 1 h in a humidified chamber. The slides were washed with 0.1-M PBS, stained in DAB solution, washed with distilled water, dehydrated in a graded ethanol series, fixed in xylene and cover-slipped in mounting medium. ImageJ software was used to quantitatively determine hyperactivated p-JNK, COX-2, HO-1, Nrf2, p-NFκB, Bcl2, and caspase3 in cortex/total area and in the striatum/total area by optimizing background of images according to the threshold intensity and analyze p-JNK, COX-2, HO-1, Nrf2, p-NFκB, Bcl2, and caspase3 positive cells at the same threshold intensity for all groups. The intensity is expressed as the relative integrated density of the samples relative to the control.

### Immunofluorescence Analysis

Slides were treated with proteinase K (antigen retrieval step), washed with 0.1 M PBS, and incubated with 5% normal serum according to the source of the secondary antibody used. The slides were incubated with primary antibodies against Nrf2 (dilution: 1:100, Santa Cruz Biotechnology), overnight at 4°C. The next morning, after being washed with PBS, the slides were incubated with fluorescently labeled secondary antibodies (dilution: 1:50, Santa Cruz Biotechnology) for signal amplification in a dark chamber, then coverslipped in Ultra Cruz mounting medium (Santa Cruz Biotechnology, Inc.). Immunofluorescence images (five images per slide) were captured using fluorescent scanning microscopes (fluorescence microscope), and the same region of the cortex/total area for all groups was quantitated as above.

### Oxidative Enzymes Analysis

Oxidative stress markers such as GSH and glutathione *S*-transferase (GST) levels were determined to assess the degree of oxidative damage and the relative effect of the testing drug. GSH was determined using a previously reported method with slight modifications (Imran et al., 2020). Test drug, phosphate buffer solution and freshly prepared DTNB solution were added together and change in the color was measured at 412 nm using a spectrophotometer. Phosphate buffer was used as blank whereas the DTNB solution was used as control. Increased absorbance of the mixture revealed the presence of GSH in the test drug. Real absorbance was calculated by subtracting the absorbance of control from that of the sample. Final GSH values were expressed in μmoles GSH/mg of sample. Likely, for the determination of GST, the previously reported protocol was followed. GSH and CDNB were mixed with the test drug and the optical density of the reaction mixture was recorded at 340 nm against phosphate buffer blank in a spectrophotometer. Assay mixture without drug served as control. Increased absorbance indicated the presence of GST and the antioxidant properties of the sample. GST activity was calculated using the extinction coefficient of the product formed and expressed as μmoles of CDNB conjugated/min/mg of protein.

### LPO Assay

Lipid peroxidation (LPO) assay was carried out by measuring thiobarbituric acid reactive substances (TBARS) using fluorimetry as described previously with slight modifications (Ullah et al., 2020). Rats forebrain was homogenized in 10 mL of 20 mM Tris-HCl (pH 7.4) at 4°C in a polytron homogenizer. Homogenate was then centrifuged at 1,000 *g* for 10 min at 4°C and supernatant was isolated. Freshly prepared ferric ammonium sulfate was then added to this supernatant (40 μL) and incubated at 37°C for 30 min. Finally, 75 μL of thiobarbituric acid (TBA) was added and absorbance was measured immediately at 532 nm using a microplate reader and expressed as Tbars-nM/min/mg protein.

### Enzyme-Linked Immunosorbent Assay (ELISA)

The Nrf2, HO-1, p-NFκB, TNF-α expression was measured using Rat Nrf2, HO-1, p-NFκB, TNF-α enzyme-linked immunosorbent assay (ELISA) kit according to the manufacturer’s instructions (for detail chemicals and reagents). The tissues (approximately 50 mg) were homogenized at 15,000 RPM using Silent Crusher M (Heidolph) and the supernatant was collected after centrifugation (at 4,000 × *g* for 10 min). The total protein concentration in each group was determined by the BCA method (Elabscience) and the equivalent quantity of protein was then loaded to determine the concentration of Nrf2, HO-1, p-NFκB, and TNF-α by using ELISA microplate reader (BioTek ELx808). and finally, the concentrations (pg/ml) were then normalized to total protein content (pg/mg total protein).

### Docking Studies

Genetic Optimization of Ligand Docking (GOLD v5.2.2) package was used for docking. The 3D structure of Keap1 (PDB ID: 3CGJ) in complex with RA839 inhibitor was taken from protein data bank (Winkel et al., 2015). All water molecules, heteroatoms, and other unwanted molecules were removed from the Keap1 structure before docking simulation. Hydrogen atoms were added and the docking site of Keap1 was traced from the bound inhibitor (RA839). The 2D structures of carveol were taken from PubChem and prepared for docking in Discovery Studio *v*4.5 (DS). During the docking protocol, a total of 100 conformational poses of carveol were generated using the *Genetic Algorithm* (GA) module of the GOLD package. Furthermore, the piecewise linear potential (ChemPLP) was used as the scoring function and the results were evaluated. The promising inhibitory pose of carveol was determined by the highest ChemPLP and polar and non-polar interactions with the crucial residues of Keap1. The docking results were analyzed using DS and GOLD software.

### Statistical Analysis

The data are presented as means ± SEM. Data were analyzed by one-way or two-way analysis of variance (ANOVA) followed by a *post hoc* Bonferroni multiple comparison test using GraphPad Prism 6 software. Moreover, behavior studies were analyzed by grouped two-way analysis. Immunohistochemical data and TTC data were analyzed using ImageJ software^1^ (ImageJ 1.30). In all analyses, differences were considered significant at *p* < 0.05. The symbol ^∗^ indicates a significant difference relative to sham, # shows a significant difference relative to MCAO.

## Results

### Effect of Post-treatment Dosage Regimen of Carveol on Brain Infarction and Neuronal Cell Loss

**FIGURE 2:**
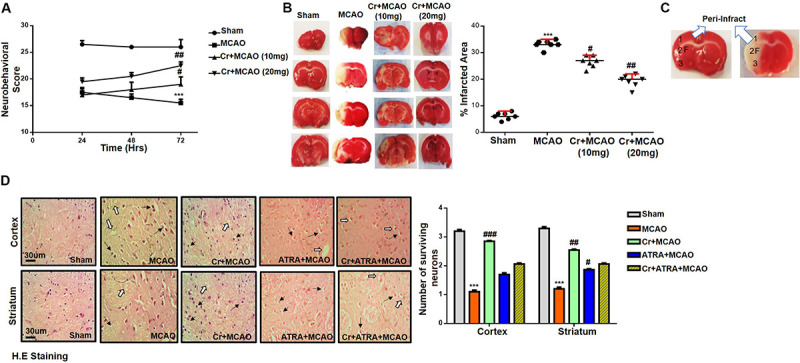
Effect of post-treatment dosage regimen of carveol on brain infarction and neuronal cell loss. **(A)** 28 points composite scoring. CR + MCAO rats had significantly reduced neurological deficits (^#^*p* < 0.05 vs ^##^*p* < 0.01) as compared to MCAO rats. Data are presented as mean ± SEM and analyzed by two-way ANOVA (group analysis) (*n* = 7/group). **(B)** Brain coronal sections stained with TTC were used for analysis. Data are presented as mean ± SEM and analyzed by one-way ANOVA (*n* = 7/group). ***p < 0.001, ^#^*p* < 0.05, and ^##^*p* < 0.01 significantly different. The symbol * shows significant difference relative to sham while # shows significant difference relative to MCAO. **(C)** Coronal sections separated by frontal cortex (1), parietal cortex and insular cortex (2), and the piriform cortex (3). The analyzed region of interests indicated by 1 and F. **(D)** Representative images of hematoxylin and eosin staining in various groups (*n* = 5/group), carveol used as 20 mg/kg and ATRA as 5 mg/kg. H.E slides were made by processing TTC thick coronal sections which were fixed in 4% paraformaldehyde. From the thick coronal TTC sections, paraffin blocks were made, and later 4 μm thin coronal sections were prepared by a rotary microtome. Arrows indicate shrunken and condensed nuclei and open arrows indicate swelled and vacuolated forms. Scale bar = 50 μm. * Significant difference relative to control; # significant difference relative to MCAO group. All data are presented as means ± SEM. Data were analyzed using two-way ANOVA followed by *post hoc* Bonferroni multiple comparison test using GraphPad Prism 6 software. ***Indicates *p* < 0.001 shows significant difference relative to sham control while, ^##^indicates *p* < 0.01 and ^#^indicates *p* < 0.05 showing significant difference relative to MCAO group.

The potential effects of carveol on the chronic pathological and neurological changes after MCAO were first examined. The neurological score was determined daily for 72 h. As shown in Figure 2A, MCAO animals exhibited severe neurological deficits as demonstrated by deficits of cortical and striatal controlling functions, such as sensory and motor coordination (Figure 2A, *p* < 0.001), which was significantly reversed by daily carveol treatment in a dose-dependent manner (Figure 2A). Specifically, 20 mg/kg carveol showed improved protective efficacy to reverse the neurological deficits after 72 h of MCAO (Figure 2A, *p* < 0.01) as compared to 10 mg/kg of carveol. TTC staining was performed to further evaluate the pathological changes and cellular viability. MCAO produced a significantly larger infarction area as compared to sham-operated group (*p* < 0.001, Figure 2B), while carveol treatment significantly attenuated the infarction in a dose-dependent manner (*p* < 0.05, *p* < 0.01, Figure 2B), with the corrected infarct area of 33.14 ± 0.59% in MCAO, 26.5 ± 0.8% at 10 mg/kg, and 19.2 ± 0.86% at 20 mg/kg in Cr + MCAO groups (Figure 2B), respectively. H&E staining was used to reveal the extent of neuronal injury in cortex and striatum 72 h after occlusion and to further examine the neuroprotective effects of carveol. The peri-infarct frontal cortex and striatum were visualized for morphological analysis Figure 2C. As shown in (Figures 2C,D), considerable variations were observed in color staining, neuronal shape, and neuronal integrity after MCAO. Further evaluation of color staining and vacuole formation indicated robust detrimental changes in the ipsilateral cortex and striatum (Figure 2D, *p* < 0.001), whereas carveol treatment attenuated the damage and increased number of intact neuronal cells could be seen (Figure 2D, *p* < 0.001).

### Effects of ATRA on Carveol Mediated Neuroprotection

**FIGURE 3:**
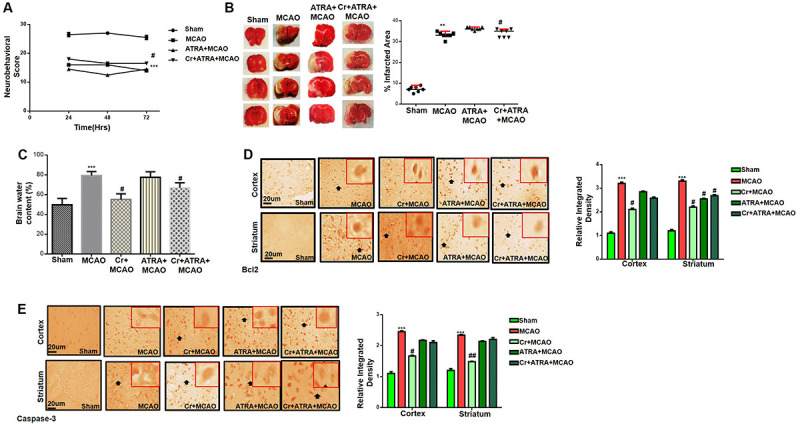
Effects of ATRA on carveol mediated neuroprotection. **(A)** 28 points composite scoring. ATRA + MCAO rats had significantly severe neurological deficits than MCAO rats had (*p* < 0.01). Neurological score data is presented as mean ± SEM and analyzed by two-way ANOVA (group analysis) (*n* = 7/group). Carveol used as 20 mg/kg and ATRA as 5 mg/kg. **(B)** Brain coronal sections were stained with TTC, which distinguishes between ischemic and non-ischemic areas (*n* = 7/group). Carveol used as 20 mg/kg and ATRA as 5 mg/kg. Data are presented as mean ± SEM and analyzed by one-way ANOVA. **p < 0.01 and ^#^*p* < 0.05. Symbols * shows significant difference relative to sham while # shows significant difference relative to MCAO. **(C)** The brain water content of the cerebral hemisphere in the ischemic side was calculated by the wet and dry method in each group (*n* = 5/group). Carveol used as 20 mg/kg and ATRA as 5 mg/kg. Data are presented as mean ± SEM and analyzed by one-way ANOVA. ***p < 0.001 relative to the sham group; ^#^*p* < 0.05 relative to the MCAO group. **(D)** Immunohistochemistry results for Bcl-2 in cortex and striatum. Scale bar 50 μm, magnification 40×. **(E)** Immunohistochemistry results for caspase-3 in cortex and striatum. Scale bar 50 μm, magnification 40× (*n* = 5/group). Carveol used as 20 mg/kg and ATRA as 5 mg/kg. Data are presented as mean ± SEM and analyzed by two-way ANOVA followed by *post hoc* Bonferroni multiple comparison test using GraphPad Prism 6 software. Both Bcl-2 and caspase-3 exhibited cytoplasmic localization in both tissues. Immuno slides were made by processing TTC thick coronal sections which were fixed in 4% paraformaldehyde. From the thick coronal TTC sections, paraffin blocks were made, and later 4 μm thin coronal sections were prepared by a rotary microtome. ***p < 0.001 shows significant difference relative to sham, ^#^*p* < 0.05, and ^##^*p* < 0.01 shows significant difference relative to MCAO group.

Several studies demonstrated the inhibitory effect of ATRA on the Nrf2 signaling pathway and different doses of ATRA was employed to inhibit Nrf2 in several experimental models (Wang X. J. et al., 2007). We then investigated whether Nrf2 could be involved in the underlying neuroprotective effects of carveol. Based on our preliminary results (data not shown), 5 mg ATRA was found to inhibit Nrf2 in our study, and this inhibition by ATRA further exacerbates the MCAO induced damage as shown (Table 1). These results were further validated via neurobehavioral defects (Figure 3A), whereas composite score was reduced to (16.8 ± 0.73) in Cr + ATRA + MCAO group like that of MCAO group (15.7 ± 0.99) (Figure 3A) and with the corrected infarct area to be (33 ± 0.59%) and (36 ± 0.50%), respectively in MCAO and Cr + ATRA + MCAO group (Figure 3B). Meanwhile, the brain water content in the MCAO group was considerably higher relative to the sham group (*p* < 0.001, Figure 3C) whereas the edema in the carveol treated group (20 mg/kg) was reduced significantly (*p* < 0.05, Figure 3C). These data suggest that carveol treatment can mitigate brain edema after t-MCAO and ATRA abrogated carveol effects, supporting the necessary role of Nrf2. To further examine whether t-MCAO induced neuronal apoptosis is accounted for this cell death at the peri-infarct region, we investigated the expression level of caspase-3 and Bcl2 in the ischemic frontal cortex and striatum (Figures 3D,E). Our results demonstrated higher caspase-3 along with a reduced expression of Bcl2 in the ipsilateral cortex and striatal tissue following 72 h after MCAO relative to the sham-operated group (Figures 3D,E). Notably, carveol treatment significantly attenuated the expression of caspase-3 (*p* < 0.05) suggesting that carveol could ameliorate the MCAO induced neuronal apoptosis.

**TABLE 1:** Carveol (20 mg/kg) ameliorated oxidative stress and neuroinflammation.

Groups	GSH (μmoles/mg of protein)	GST (μmoles CDNB conjugate/min/mg of protein)	Catalase (μmoles H202/min/mg of protein)	LPO(Tbras-nM/min/mg protein)
Sham	60.7 ± 0.84	43.2 ± 1.62	25.0 ± 2.61	40.5 ± 0.42
MCAO	13.65 ± 0.48***	10.75 ± 2.05***	8.22 ± 0.96^∗∗∗^	119.8 ± 0.73^∗∗∗^
Cr + MCAO	41.4 ± 1.83^###^	27.9 ± 3.11^#^	17.1 ± 0.82^##^	63.8 ± 1.05^###^
ATRA + MCAO	20 ± 1.97	15.6 ± 1.83	7.10 ± 1.04	112.3 ± 0.52
Cr + ATRA + MCAO	17.8 ± 2.24	13.7 ± 2.06	9.1 ± 1.15	96.3 ± 0.73

Carveol significantly normalized the cortex antioxidant enzyme levels. Symbols *** shows significant difference *p* < 0.001 while symbol ## represents *p* < 0.01 values for significant difference. All data were presented as means ± SEM and analyzed by one-way ANOVA followed by *post hoc* Bonferroni multiple comparison test. Symbols * show a significant difference of MCAO relative to sham and # shows a significant difference of MCAO (*n* = 5/group). After euthanasia, samples were collected and preserved at −80°C. Abbreviations: GST, glutathione *S*-transferase; GSH, glutathione; TBARS, thiobarbituric acid reactive substances; LPO, lipid peroxidation. ^###^Indicates *p* < 0.001 and it shows significant difference relative to MCAO.

### Carveol Ameliorated Oxidative Stress and Neuroinflammation

**FIGURE 4:**
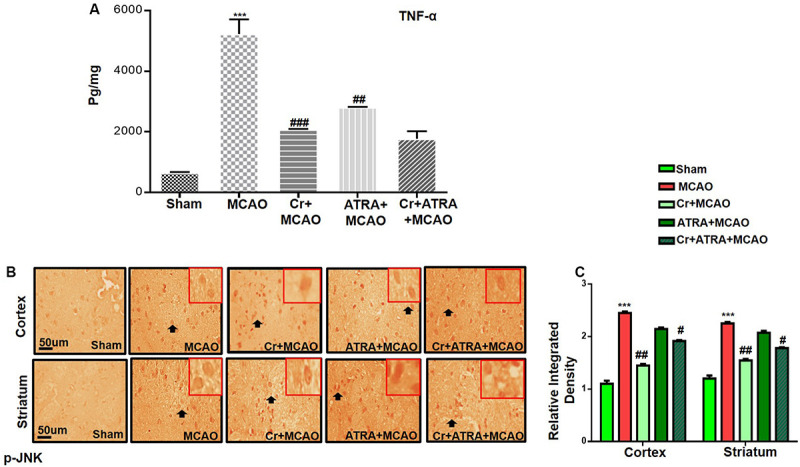
Carveol (20 mg/kg) ameliorated oxidative stress and neuroinflammation. **(A)** TNF-α expression was quantified by ELISA in the cortex. The data were expressed as the mean ± SEM and analyzed by one-way ANOVA. ***p < 0.001 relative to the sham while ^##^*p* < 0.01 relative to the MCAO with *n* = 5/group. After euthanasia, samples were collected and preserved at –80°C and later processed for protein analysis. **(B)** Immunohistochemistry results for pJNK in cortex and striatum tissues of the brain. Scale bar 50 μm, magnification 40× (*n* = 5/group). p-JNK exhibited cytoplasmic localization in both tissues. Histograms show a comparatively higher expression of p-JNK in various segments of the MCAO and ATRA groups. The data were expressed as the mean ± SEM and analyzed by two-way ANOVA followed by *post hoc* Bonferroni multiple comparison test using GraphPad Prism 6 software. ***p < 0.001 shows significant difference relative to sham, while ^#^*p* < 0.05, ^##^*p* < 0.01 and ^###^*p* < 0.001 shows significant difference relative to MCAO group. Immuno slides were made by processing TTC thick coronal sections which were fixed in 4% paraformaldehyde. From the thick coronal TTC sections, paraffin blocks were made, and later 4 μm thin coronal sections were prepared by a rotary microtome.

Oxidative stress and inflammation are two interrelated pathophysiological processes involved in ischemic stroke that are early initiated and exhibited as a prolonged detrimental effect until the recovery stage. TBARS is a commonly used method to measure lipid peroxidation end product malondialdehyde, a reactive aldehyde produced by lipid peroxidation of polyunsaturated fatty acids. We then performed the TBARS test and the results showed a drastic increase of peroxides in the MCAO group, an effect that could be rescued by carveol treatment (Table 1). The LPO content in the cortical homogenate was increased to (119.8 ± 0.73) in the MCAO group as compared to (40.5 ± 1.05) in the sham-operated group (*p* < 0.001, Table 1). A total of 20 mg/kg carveol significantly (*p* < 0.05, Table 1) attenuated this increase (63.14 ± 1.05) to a level comparable to that of the sham-operated group. We then examined the effects of carveol on inflammatory-related factors. As shown in Figure 4, a higher level of TNF-α could be observed after MCAO, which could be attenuated by carveol treatment (*p* < 0.001, Figure 4A) by ELISA analysis. Similarly, JNK, one of the major signaling of the mitogen-activated protein kinase (MAPK) pathway which functions in the control of apoptosis, inflammation, cytokine production, and metabolism, showed a reduced expression after carveol treatment, demonstrating the antioxidative and anti-inflammatory effects of carveol (*p* < 0.01, Figure 4B).

### Carveol Enhances the Antioxidant Capacity of the Brain via the Nrf2 Signaling Pathway

**FIGURE 5:**
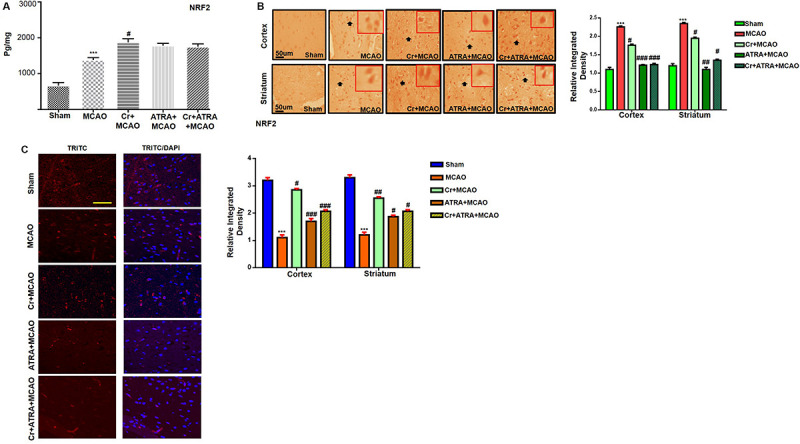
Carveol (20 mg/kg) enhances the antioxidant capacity of the brain via the Nrf2 signaling pathway. **(A)** The protein expression of Nrf2 was quantified by ELISA in cortical tissue. The data were expressed as the mean ± SEM and were analyzed by one-way ANOVA. ***p < 0.001 shows difference relative to sham while ^#^*p* < 0.05 shows significant difference relative to MCAO with *n* = 5/group. After euthanasia, samples were collected and preserved at –80°C and later processed for protein analysis. **(B)** Immunohistochemistry results for Nrf2 in cortex and striatum tissues of the brain. Scale bar 50 μm, magnification 40× (*n* = 5/group). Nrf2 exhibited nucleus localization in both tissues. Histograms show a comparatively higher expression of Nrf2 in various segments of the MCAO and CR groups. The data were expressed as the mean ± SEM and were analyzed by two-way ANOVA. ***p < 0.001 shows significant difference relative to sham, while ^#^*p* < 0.05, ^##^*p* < 0.01 and ^###^*p* < 0.001 shows significant difference relative to MCAO group. **(C)** Immunofluorescence reactivity of Nrf2 in the cortex (*n* = 5/group). The above data is a representation of three numbers of experiments. Scale bar = 100 mm and magnification 40×. Nrf2 was visualized by Alexa-Flour 594. Data are presented as means ± SEM. Data is analyzed by one-way ANOVA followed by *post hoc* Bonferroni multiple comparison test using GraphPad Prism 6 software.

**FIGURE 6:**
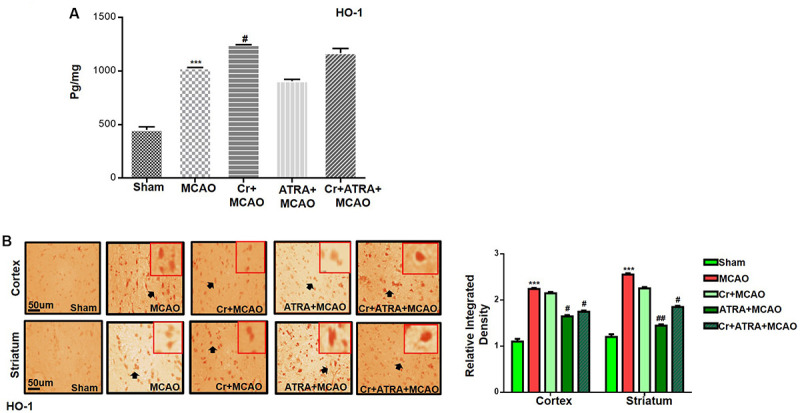
Carveol (20 mg/kg) enhances the antioxidant capacity of the brain via the Nrf2 signaling pathway. **(A)** ELISA analysis of HO-1 in the cortex. The data were expressed as the mean ± SEM and analyzed by one-way ANOVA followed by *post hoc* Bonferroni multiple comparison test using GraphPad Prism 6 software. ***p < 0.001 shows difference relative to sham while ^#^*p* < 0.05 shows significant difference relative to MCAO. **(B)** Immunohistochemistry results for HO-1 in cortex and striatum tissues of the brain. Scale bar 50 μm, magnification 40× (*n* = 5/group). HO-1 exhibited nucleus localization in both tissues. Histograms show a comparatively higher expression of Nrf2 in various segments of the MCAO and CR groups. ***p < 0.001 shows significant difference relative to sham, while ^#^*p* < 0.05 and ^##^*p* < 0.01 shows significant difference relative to MCAO group.

Nuclear factor erythroid 2-related factor 2 (Nrf2) is an endogenous antioxidant enzyme which executes vital protective functions (Reisman et al., 2009). Translocation of Nrf2 to nucleus imitates transcription of several downstream antioxidant proteins like heme oxygenase-1 (HO-1), superoxide dismutase (SOD), and GSH to annihilate ROS and protect the cell from inflammation and apoptosis (Ning et al., 2018). Nrf2 also suppressed the expression of proinflammatory factors like p-NFκB and COX2. Thus, to examine the possible effect of carveol on Nrf2 related antioxidant signaling pathway and pro-inflammatory factors, we measured their expression levels. As shown by ELISA results in Figure 5A, Nrf2 expression was increased in the MCAO group, while carveol treatment further increases their expression (*p* < 0.05). To further validate our ELISA results, we performed immunohistochemical analysis (Figure 5B) and immunofluorescence analysis (Figure 5C). Likely, HO-1 (Figure 6) GST and GSH showed a similar pattern of changes. The changes in antioxidative enzyme levels of different groups were also summarized in Table 1. MCAO induced an elevation in ROS generation, associated with depletion of GSH level (13.65 ± 0.48), GST activity (10.75 ± 2.05), and catalase (8.22 ± 0.96) in the brain cortical tissue (*p* < 0.001). Treatment with carveol attenuated downregulation of GSH (41.4 ± 1.83), GST (27.9 ± 3.1), and catalase (17.1 ± 0.82), which is comparable to the sham-operated group.

### Effect of Carveol on Outcomes of MCAO Induced Inflammatory Mediators

**FIGURE 7:**
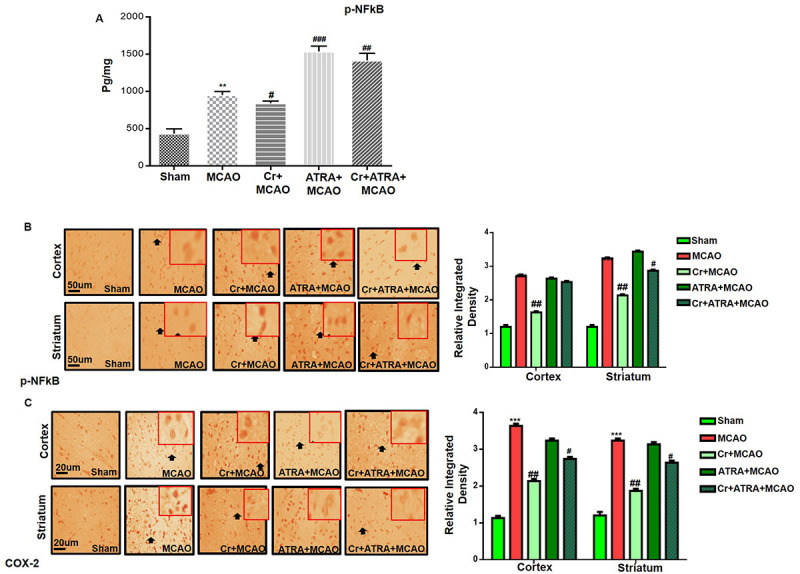
Effect of carveol (20 mg/kg) on outcomes of MCAO induced inflammatory mediators. **(A)** The protein expression of p-NFκB was quantified by ELISA in the brain cortex. The data were expressed as the mean ± SEM and were analyzed by one-way ANOVA with *n* = 5/group. **p < 0.001 shows difference relative to sham while ^#^*p* < 0.05, ^##^*p* < 0.01, and ^###^*p* < 0.001 shows significant difference relative to MCAO. **(B)** Immunohistochemistry results for p-NFκB in cortex and striatum tissues of the brain. Scale bar 50 μm, magnification 40×. **(C)** Immunohistochemistry results for COX-2 in the cortex and striatum tissues of the brain. Scale bar 50 μm, magnification 40× (*n* = 5/group). p-NFκB exhibited nucleus localization while COX-2 exhibited cytoplasmic localization in both tissues of the brain. Histograms show comparatively higher expression of p-NFκB and COX-2 in various segments of the MCAO and ATRA groups. ***p < 0.001 shows significant difference relative to sham, while ^#^*p* < 0.05 and ^##^*p* < 0.01 shows significant difference relative to MCAO group. Data are presented as means ± SEM. Data is analyzed by two-way ANOVA followed by *post hoc* Bonferroni multiple comparison test using GraphPad Prism 6 software. Immuno slides were made by processing TTC thick coronal sections which were fixed in 4% paraformaldehyde. From the thick coronal TTC sections, paraffin blocks were made and later 4 μm thin coronal sections were prepared by a rotary microtome.

Pro-inflammatory factors TNF-α and IL-1β binding to respective receptors triggered sequential activation of downstream molecules such as ASK1, SEK1, and JNK. Collectively, this leads to proteasomal dependent IκB dissociation, and nuclear translocation of NF_*K*_B to induce inflammatory transcription types of machinery like NOS2 and COX2 (Davies et al., 2016). These proteins were hyper expressed in ELISA findings in the ischemic brain (*p* < 0.01, Figure 7A), and the carveol post-treatment dosage regimen significantly alleviated the expression (*p* < 0.05). These results were further validated by immunostaining findings for p-NF-κB, and COX2 (Figures 7B,C).

### Bioinformatics Analysis

**FIGURE 8:**
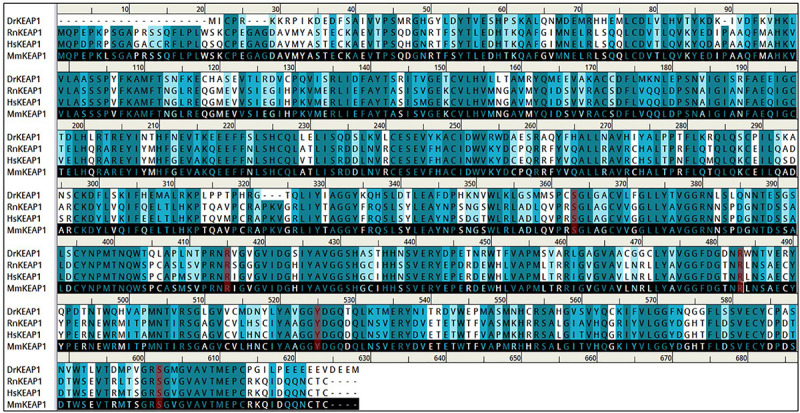
Arg483, Ser363, Arg525, and Ser602 are conserved residues in the Nrf2-binding site of Keap1.

**FIGURE 9:**
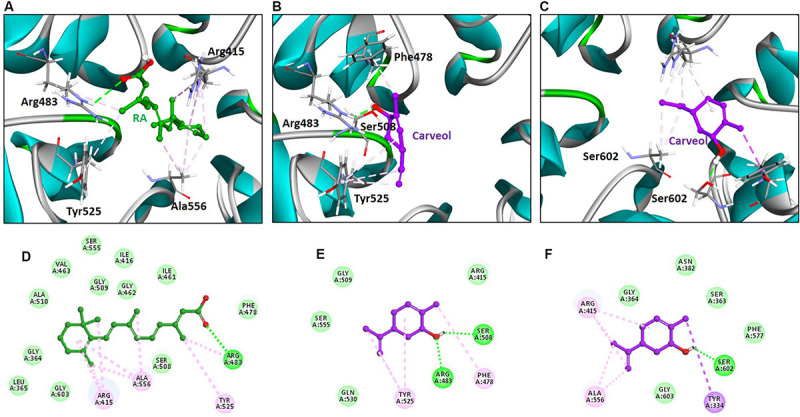
Retinoic acid and carveol occupy the Nrf2-binding site of Keap1. The upper panel **(A–C)** shows the binding orientation of retinoic acid and carveol in the Nrf2-binding site of Keap1. **(A)** Retinoic acid established hydrogen bond with Arg483 of Keap1. **(B)** One carveol molecule formed a hydrogen bond with Arg83 and Ser508 in the acidic region of Keap1. **(C)** Another carveol molecule established a hydrogen bond with Ser602 in the sulfamide region of Keap1. Retinoic acid and carveol are represented as ball and stick models and colored as green and magenta, respectively. Keap1 residues participated in either polar or non-polar interactions are depicted as stick representation. Hydrogen bonds are represented as green color dashed lines. Other hydrophobic interactions are portrayed as light magenta-colored dash lines. Lower panel **(D–F)** displayed the 2D (two dimensional) interaction pattern of Retinoic acid and carveol with Keap1. **(D)** Retinoic acid, **(E)** carveol in the acidic region of Keap1, and **(F)** carveol in the sulfamide region of Keap1 formed hydrogen bonds with Keap1. Retinoic acid and carveol are presented as ball and stick models and colored as green and magenta, respectively. Hydrogen bonds forming residues are represented as green color circles and labeled. Hydrogen bonds are displayed as green-colored dash lines. Other non-polar interacting residues are depicted as light green colored (residues forming Van der Waals interactions) and light magenta-colored (hydrophobic interactions) and labeled.

The above experimental results suggested that carveol activates Nrf2 signaling, which is localized to the cytoplasm as an inactive dimer with keap1. Therefore, we then performed docking studies of carveol and Keap1 to examine the possibility that carveol may bind Keap1 to disrupt its interaction with Nrf2 and facilitate its migration to the nucleus. The docking results demonstrated that carveol could bind Keap1 and orient in the Nrf2 binding site. Since the Nrf2 binding site of Keap1 is shallow and sub-divided into the acidic region, the planner acceptor region and sulfamide pocket (Figure 8) (Winkel et al., 2015). Therefore, we asked the suitable sub-site and binding orientation of carveol in Keap1. Our results demonstrated that carveol oriented in two different stoichiometric conformations in the Nrf2-binding site of Keap1 (Figures 9B,C). Carveol occupied the acidic region (Figure 8B) and sulfamide pocket (Figure 8C) of the Nrf2-binding site of Keap1 and obtained a docking score of 33.88 and 35.18, respectively (Table 2).

**TABLE 2:** Docking scores and hydrophobic interactions of retanoic acid and carveol with Keap-1.

Inhibitor	Docking score	Hydrogen bonds (<3.5 Å)				Hydrophobic interactions
		Amino acid	Amino acid atom	Ligand atom	Distance (Å)	
Retanoic Acid	54.19	Arg483	HE	02	3.0	Arg415, Ala556, Tyr525, Ser508, Phe478, Ile461, Gly462, Ile416, Gly509, Ser555, Val463, Ala510, Gly364, Leu365, Gly603
Carveol A*	33.88	Arg483 Ser508	HE OG	O1 H25	2.0 1.9	Tyr525, Phe478, Arg415, Gly509, Ser555, Gln530
Carveol S^#^	35.18	Ser602	OG	H25	1.7	Arg415, Ala556, Tyr334, Gly603, Phe577, Ser363, Asn382, Gly364

*Carveol A: Carveol in the acidic region. ^#^Carveol S: Carveol in sulfamide region. Abbreviations: Gly, glycine; Ala, alanine; Ser, serine; Tyr, tyrosine; Ile, iso-leucine; Arg, arginine; Phe, phenylalanine; Val, valine; Leu, leucine; Gln, glutamine.

Furthermore, the molecular interaction analysis suggested that carveol established hydrogen bond interaction with Arg483 in the acidic region of Keap1 (Figures 9B,E). In the acidic region of Keap1, carveol also formed another hydrogen bond with Ser508 (Figures 9B,E). Moreover, the molecular interaction analysis of carveol in the sulfamide region established a hydrogen bond with Ser 602 of Keap1 (Figures 9C,F). Collectively, we speculate that carveol may orient in the acidic as well as sulfamide sub-pockets of Keap1 and forms hydrogen bonds with Arg483 and Ser602, respectively. Apart from the hydrogen bond formed with key residues of Keap1, carveol could also establish non-polar interactions with Nrf2 binding residues of Keap1 (Figure 9, Table 2). For instance, Tyr525 formed hydrophobic interaction with carveol which has also been reported for other small molecules inhibitors (Marcotte et al., 2013). In the sulfamide region, carveol formed π–σ interaction with Tyr334 and alkyl interactions with Arg415 and Ala556 (Figures 9C,F). Apart from polar interaction with Arg415, carveol established several non-polar (hydrophobic and Van der Waals interactions) with the Nrf2 binding site residues of Keap1 (Figure 9, Table 2). Therefore, we demonstrate that carveol occupies the Nrf2 binding site of Keap1 and reduces the binding frequency of Nrf2 and Keap1.

## Discussion

**FIGURE 10:**
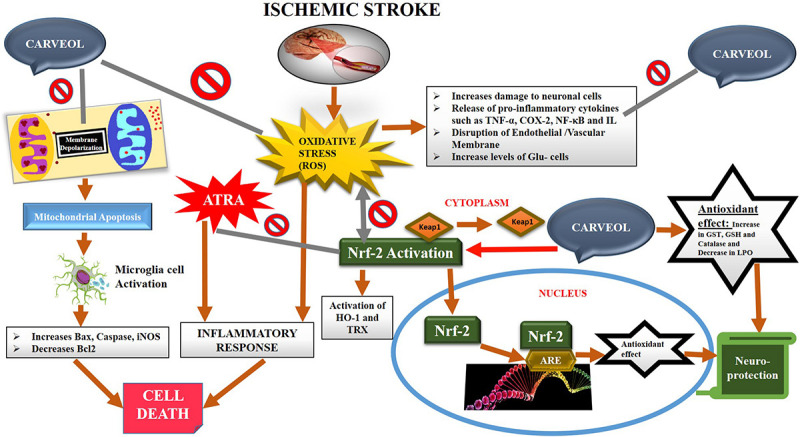
The graphical representation indicates and elaborates on the underlying antioxidant and anti-inflammatory mechanisms of carveol against the MCAO-induced brain injury.

The natural drug substances are significantly investigated for their therapeutic potentials including in various neurodegenerative models both to understand the pathophysiology and to develop better therapeutic options. In the present study, our objective is to investigate for the first time (as per our knowledge and literature survey) the neuroprotective effects of naturally derived substance carveol against MCAO induced oxidative stress and neurodegeneration. Carveol attenuated MCAO induced apoptosis and neurodegeneration both in cortical and striatal tissues after 72 h of transient ischemic injury. These beneficial and neuroprotective effects are likely mediated by mitigating neuroinflammation, oxidative stress, and enhancing free radical scavenging activity. Further, our results demonstrated that carveol act as a potent antioxidant and modulator of Nrf2, which stimulated the other antioxidative mechanism, including GSH and HO-1, and reducing pro-neuroinflammatory factors and mediators such as p-NF-κB (Figure 10).

Post-ischemic treatment reveals a more valuable and clinically significant approach both for understanding the pathophysiology and to develop better therapeutic options over a longer period. Excessive accumulation of ROS is an established and well-studied pathological mechanism in experimental ischemic stroke (Kurian et al., 2016), and which is further associated with different cellular signaling, implicated in neuroinflammation and thus further intensifies the pathogenesis of ischemic injury (Aguilera et al., 2018; Solleiro-Villavicencio and Rivas-Arancibia, 2018; Kishimoto et al., 2019). Therefore, oxidative stress is a rational avenue to investigate the anti-oxidative potential of natural drug substances (Li and Yang, 2016; Yang et al., 2019). Our results showed that carveol reduced the elevated oxidative stress, coherent with the previous study, whereas the carveol has been reported to exhibit antioxidant and anti-hyperlipidemic activities (Johri, 2011). Further, we assessed that this anti-oxidative activity of carveol may be attributed to Nrf2 activation and its downstream HO-1 targets which subsequently regulate and mobilize other endogenous antioxidant systems, such as SODs, GSH, and sirtuins (Sirt). In our study, antioxidant activity in the ischemic cortex was assessed and increased levels of GST, GSH, and catalase were observed associated with reduced LPO level, illustrating its antioxidant nature in our ischemic model. The detailed molecular mechanisms of carveol effects are still unidentified but could be partially attributed to its free radical scavenging properties. Previous studies reveal that the Nrf2 network plays a central role in the cellular adaption by dealing with a wide range of cytoprotective proteins, counteracting distinct endogenous and exogenous insults, and providing a promising optimal therapeutic target against multiple diseases from cancer to vascular and brain disorders (Dinkova-Kostova et al., 2018; Sivandzade et al., 2019; Panieri and Saso, 2019; Brandes and Gray, 2020). The role of the Nrf2 in ischemic stroke and its underlying neuroprotection has also been evidenced using different ischemic stroke rodent models (Wang X. J. et al., 2007; Aguilera et al., 2018; Solleiro-Villavicencio and Rivas-Arancibia, 2018; Kishimoto et al., 2019).

The oxidative stress associated kinases such as activated p-JNK (MAPK) signaling has a pivotal role in neuroinflammation and apoptotic neurodegeneration (Choudhury et al., 2015; Chen et al., 2018). Numerous studies reported that suppression and inhibition of p-JNK could attenuate neuroinflammation and neurodegeneration not only in ischemic stroke but also in other neurodegenerative models (Atochin et al., 2016; Zheng et al., 2020). Interestingly, here we also found that carveol downregulated the activated p-JNK in the ischemic brain compared to the sham control, which described that the carveol neuroprotective effect may be partially associated with the regulation of MAPKs.

A strong relationship between inflammation and oxidative stress is revealed in both neuronal and non-neuronal models (Yeligar et al., 2010; Ali et al., 2018). The release of inflammatory factors (IL-1b and IL-6) are abruptly enhanced at least 48 h after MCAO (Clausen et al., 2017). Other than these elevated pro-inflammatory cytokines, inducible pro-inflammatory chemokines such as COX-2, NOS-2, and NF-κB is also involved in the inflammation pathology of MCAO injury (Itoh and Yamamoto, 2005; McNeill et al., 2015; Kobayashi et al., 2016). Our ELISA results suggest that carveol reduced the expression of TNF-α, COX-2, and NF-κB, associated with an increase in nuclear translation of Nrf2, and induces further other antioxidant genes. Collectively, these processes attenuate inflammation by inhibiting the NF-κB pathway and other pro-inflammatory markers, which can persist for at least 3 days after ischemic injury, as revealed by the 1.8- to 3.6-times increased levels of HO-1, NQO1, SOD2, and GPx proteins after MCAO (Liu et al., 2018). Our results that ATRA treatment abolished the neuroprotective effects of carveol further supported the pivotal role of Nrf2 as a modulator of both oxidative stress and neuroinflammation. In addition, our results are coherent with the previous observation which supported the key role of Nrf2 in the neuroinflammation. The Nrf2 overexpression or pharmacological activation rescued neuroinflammation while inhibition of Nrf2 escalated release of pro-inflammatory markers and neuroinflammation in several ischemic and other neurodegenerative models (Kobayashi et al., 2016; Yang et al., 2017; Liu et al., 2018; Ya et al., 2018; Bahn et al., 2019).

Molecular docking results indicated that carveol may competitively bind Keap1. The formation of polar and non-polar interactions between the small molecule inhibitors and the Nrf2 binding site residues of Keap1 have already investigated (Marcotte et al., 2013; Jnoff et al., 2014; Winkel et al., 2015; Davies et al., 2016; Heightman et al., 2019). Recently, Heightman et al. (2019) identified aryl propionic acid inhibitors that occupy the Nrf2-binding site of Keap1 and form hydrogen bonds with Arg483, Ser602, Ser555, and Gln530. The formation of the hydrogen bond between the small molecule inhibitor and Ser602 has also been reported (Davies et al., 2016) as well as between the small molecule inhibitor (RA839) with Arg483 and Ser508 (Winkel et al., 2015). Although the question of whether carveol could bind to these sites still merit further validation, the small size of carveol enables its conformational freedom and can preferentially orient in the shallow binding site of Keap1. Actually, such stoichiometric conformations of small molecule inhibitors have been reported for cpd15A [2-[(5-((2,5-dimethylbenzene)sulfonyl)-6-oxo-1,6-dihydropyrimidine-2-yl) sulfanyl]-*N*-(2-(trifluoromethyl)phenyl)acetamide], cpd15B and cpd16 with Keap1 (Marcotte et al., 2013). Not alone for carveol, Davies et al. (2016) has also confirmed that small molecule inhibitors bind the Nrf2-binding site of Keap1 and potentially facilitate the migration of Nrf2 into the nucleus. Previously, Jnoff et al. (2014) identified a small molecule inhibitor (1S,2R)-2-[(1S)-1-[(1,3-dioxo-2,3-dihydro-1H-isoindol-2-yl)methyl]-1,2,3,4-tetrahydroisoquinoline-2-carbonyl]cyclohexane-1-carboxylicacid [(S,R,S)-1a], which could target the Keap1-Nrf2 interaction and occupied the Nrf2-binding site of Keap1.

## Conclusion

In summary, our *in vivo* and molecular docking findings demonstrated that carveol acted as a potent antioxidant agent that triggered activation of principal endogenous antioxidant Nrf2 and ameliorated MCAO induced oxidative stress and neuroinflammation possibly by modulating the p-JNK and other neuroinflammatory mediators. We propose that carveol treatment could be an appropriate therapeutic option to reverse ischemic stroke-induced neuroinflammation and neurodegeneration. Moreover, further research work is required to completely understand the underlying protective mechanism as well as complete pharmacological and pharmacodynamic profile of carveol against stroke-induced neuroinflammation and neurodegeneration.

## Data Availability Statement

All datasets generated for this study are included in the article/Supplementary Material.

## Ethics Statement

The animal study was reviewed and approved by Institutional Animal Care and Use Committee of Riphah Institute of Pharmaceutical Sciences.

## Author Contributions

IM: conceptualization. FS: methodology, investigation, and funding acquisition. TA, NU, and IM: validation. FS and IM: resources. IM, FS, SL, and AK: writing. FS, TA, AA, and RA: review and editing. FS, ZT, and SL: supervision. All authors contributed to the article and approved the submitted version.

## Conflict of Interest

The authors declare that the research was conducted in the absence of any commercial or financial relationships that could be construed as a potential conflict of interest.

## Footnotes

1 https://imagej.nih.gov/ij/
