# Supplementary figures and images for: Potent Natural Antioxidant Carveol Attenuates MCAO-Stress Induced Oxidative, Neurodegeneration by Regulating the Nrf-2 Pathway
Source: Front Neurosci. 2020 Jun 26;14:659. doi: 10.3389/fnins.2020.00659 (PMC7344277; doi:10.3389/fnins.2020.00659)

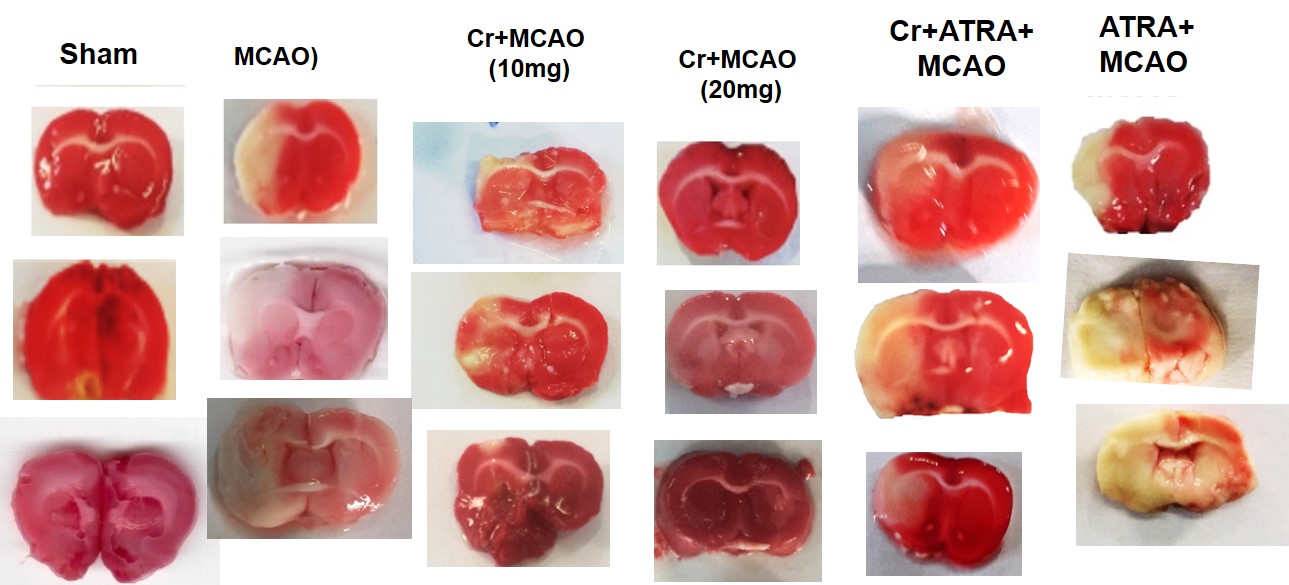

Supplement: Supplementary file 1 [file Image_1.jpeg]
